# Supplementary material for: Transcription Factor CREB3L1 Regulates the Expression of the Sodium/Iodide Symporter (NIS) in Rat Thyroid Follicular Cells
Source: Cells. 2022 Apr 13;11(8):1314. doi: 10.3390/cells11081314 (PMC9029047; doi:10.3390/cells11081314)
Supplement: Supplementary file 1 [file cells-11-01314-s001.zip › cells-1634358-supplementary.pdf]

Supplementary Figure S1

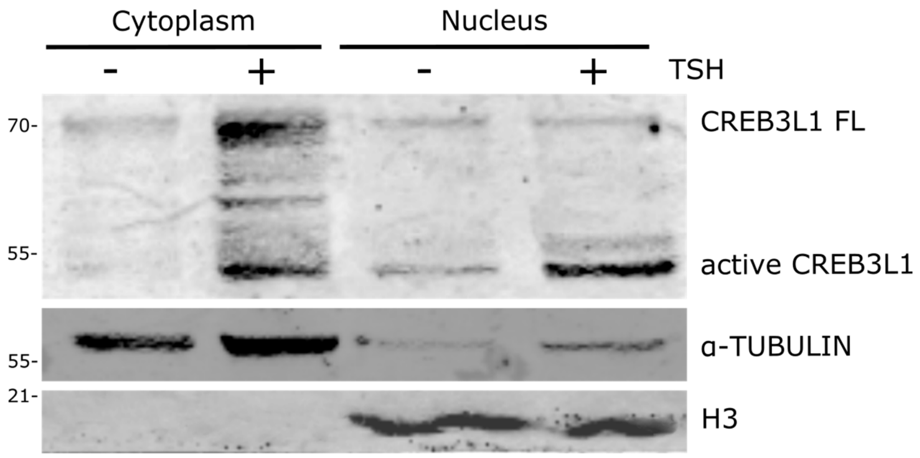

Figure S1. CREB3L1 detection in nuclear and cytoplasmic fraction of FRTL-5 cells in response to TSH: Representative western blot of cytoplasmic and nuclear extracts (see Materials and Methods) from FRTL-5 cells incubated under starvation conditions for 72 h (- TSH) and then stimulated with TSH (1.5 mIU/ml) for 16 h (+TSH). Labels on the left side indicate the molecular weight (in kDa). Histone 3 (H3) and α-tubulin were used as loading controls for the nuclear and cytoplasmic fraction, respectively.

Supplementary Figure S2

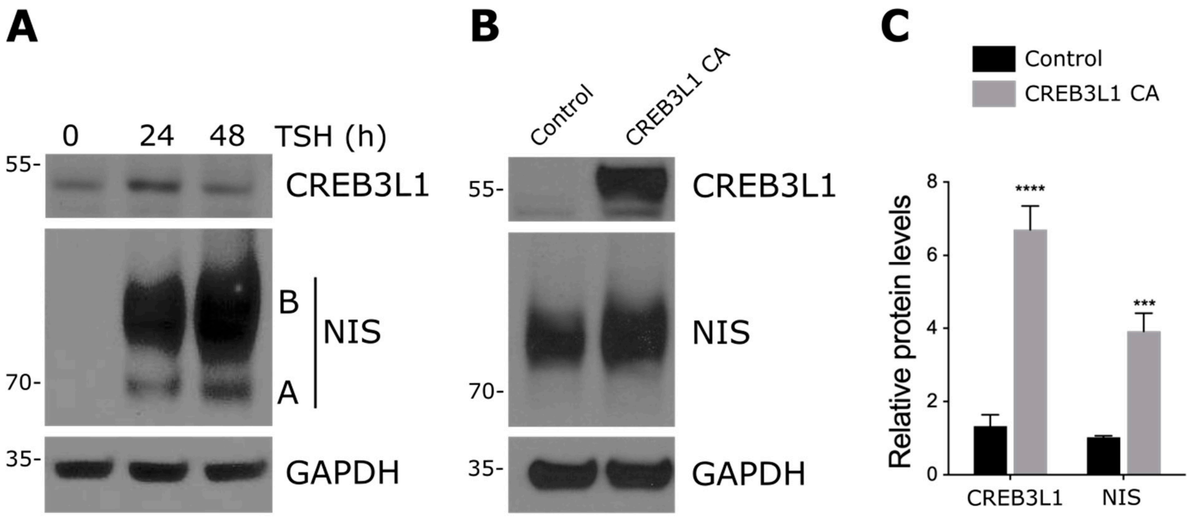

Figure S2. CREB3L1 regulates NIS expression in PCCL3 cells: (A) Representative western blot of lysates from PCCL3 cells incubated under starvation conditions for 72 h (TSH 0 h) and then stimulated with TSH (1.5 mIU/ml) for the indicated times. CREB3L1 and NIS antibodies were used. Labels on the right side of the blot indicate the relative electrophoretic mobilities of the corresponding NIS polypeptides depending on their glycosylation status: immature glycosylated (~60 kDa, band A) and fully glycosylated (~100 kDa, band B). GAPDH was used as loading control. (B) Representative western blot of lysates from PCCL3 cells, incubated under growth conditions, transiently transfected with CREB3L1 CA vector containing the N-terminus domain of CREB3L1. Control cells were transiently transfected with pcDNA 3.1 vector. (C) Densitometric quantification of proteins shown in B. The intensity of each band relative to GAPDH (loading control) was measured, values represent fold change relative to control cells. Results are presented as mean ± SEM of three independent experiments. (***P<0.001; ****P<0.0001).
